# Multiscale magnetization in cobalt-doped ferrite nanocubes^1^

**DOI:** 10.1107/S1600576722010287

**Published:** 2022-12-01

**Authors:** Dominika Zákutná, Anne Fischer, Dominique Dresen, Daniel Nižňanský, Dirk Honecker, Sabrina Disch

**Affiliations:** a Department of Chemistry, Universität zu Köln, Köln, Germany; b Faculty of Science, Charles University, Prague, Czech Republic; c ISIS Neutron and Muon Source, Rutherford Appleton Laboratory, Didcot, United Kingdom; Technical University of Munich, Munich, Germany

**Keywords:** magnetic small-angle neutron scattering, nanoparticles, ferrite, coercivity, Mössbauer spectroscopy, near-surface spin disorder

## Abstract

The magnetization of cobalt ferrite nanocubes of similar size, but with varying Co/Fe ratio, is extensively characterized on atomistic and nanoscopic length scales. Combination of X-ray diffraction, Mössbauer spectroscopy, magnetization measurements and polarized small-angle neutron scattering (SANS) reveals that a lower amount of cobalt leads to an enhanced magnetization. At the same time, magnetic SANS confirms no or negligible near-surface spin disorder in these highly crystalline, homogeneously magnetized nanoparticles, resulting in an exceptionally hard magnetic material with high coercivity.

## Abstract 2

This article presents a set of cuboidal cobalt ferrite nanoparticles with exceptionally high crystallinity, revealed by homogeneous magnetization with negligible near-surface spin disorder as observed by magnetic small-angle neutron scattering.

## 1. Introduction

The success of magnetic nanoparticles for application in biomedicine and further technologies depends critically on their dynamic magnetic properties, which can be addressed through their magnetic anisotropy by a targeted synthesis. For cancer treatment by hyperthermia, for instance, a superparamagnetic state with high saturation magnetization is highly desirable (Sathya *et al.*, 2016 ▸; Mohapatra *et al.*, 2018 ▸). In contrast, a blocked state with a large coercive field, medium magnetization and high Curie temperature is essential for application towards high-density storage (Wu *et al.*, 2014 ▸). These relevant parameters can be fine-tuned by different approaches, such as synthesizing less crystalline nanoparticles and introducing surface effects or intra-particle disorder (Lak *et al.*, 2021 ▸), replacing the cations in the nanomaterial structure to introduce different exchange coupling, or changing the morphology to induce shape anisotropy. For nanoparticles with a cuboidal shape, exceptional magnetic heating performance is reported compared with spherical nanoparticles of similar size (Guardia *et al.*, 2012 ▸), a phenomenon that can be related to enhanced magnetic surface anisotropy and facilitated face-to-face alignment into linear aggregates (Martinez-Boubeta *et al.*, 2013 ▸).

Cobalt ferrite (CoFe_2_O_4_) is particularly impressive among magnetic materials due to its high magnetocrystalline anisotropy and large coercive field (Sharrock, 1989 ▸). Nevertheless, a large amount of cobalt inside the particles is discouraged, especially for biomedical application due to its toxicity (Sanpo *et al.*, 2014 ▸). Therefore, in recent years, attention has shifted towards cobalt-deficient ferrite nanoparticles (Co_*x*_Fe_3−*x*_O_4_), which exhibit improved magnetic properties, *i.e.* increased coercivity, reduced magnetoresistance and increased saturation (Sathya *et al.*, 2016 ▸; Fantechi *et al.*, 2012 ▸). The exact composition of Co_*x*_Fe_3−*x*_O_4_ can be finely tuned by adjusting the molar ratio of Co^II^/Fe^III^ acetylacetonate precursors (Sathya *et al.*, 2016 ▸; Fantechi *et al.*, 2015 ▸, 2012 ▸; Wu *et al.*, 2014 ▸). Despite the considerable interest in Co_*x*_Fe_3−*x*_O_4_ nanoparticles, the detailed principles that modify magnetic properties are debated. The Co_*x*_Fe_3−*x*_O_4_ structure has been introduced as either a cobalt-doped magnetite structure (Fantechi *et al.*, 2015 ▸; Hu *et al.*, 2012 ▸) or a cobalt-doped maghemite structure (Fantechi *et al.*, 2012 ▸; Mohapatra *et al.*, 2018 ▸), but a clear attribution to either case is currently missing.

Considering the large body of literature on cobalt ferrite nanoparticles, a consistent picture of the evolution of the magnetic properties with Co^2+^ content is still elusive. This is surely related to the interplay of many different parameters, such as cation distribution in the spinel structure (Salazar-Alvarez *et al.*, 2007 ▸; Le Trong *et al.*, 2013 ▸), precise particle morphology (Sathya *et al.*, 2016 ▸), size and size distribution, and surface disorder (Torres *et al.*, 2015 ▸). Therefore, a targeted study of the local (atomistic) and nanoscale structure and magnetism is required to enable a rational synthesis of cobalt ferrite nanoparticles with tunable magnetic properties.

In this work, we unravel the magnetic properties of Co_*x*_Fe_3−*x*_O_4_ nanoparticles with cuboidal shape and narrow size distribution at the microscopic level, by means of half-polarized small-angle neutron scattering (SANS) in combination with Mössbauer spectroscopy and macroscopic magnetization measurements. We also precisely attribute the effect of shape and strain on the intraparticle magnetization distribution, and confirm the structural and magnetic homogeneity of our samples. We further describe the cation distribution within the spinel structure and establish a reduced spinel inversion with cobalt occupancy. The nanoparticles show enhanced coercivity as they combine high crystallinity with enhanced magnetocrystalline anisotropy through non-stoichiometric doping of cobalt.

## 2. Experimental

### 2.1. Synthesis

The cobalt ferrite nanoparticles were synthesized following thermal decomposition of cobalt and iron acetylacetonates (Wu *et al.*, 2014 ▸). In brief, 282.54 mg (0.8 mmol) of Fe(acac)_3_ and different amounts of Co(acac)_2_ [AH10: 118.29 mg (0.46 mmol); AH14: 133.72 mg (0.52 mmol); AH15: 149.15 mg (0.58 mmol)] were dispersed in 10 ml of benzylether. Sodium oleate was freshly prepared by adding 1.057 ml (3 mmol) of oleic acid to a solution of 120 mg (3 mmol) of sodium hydroxide in 1 ml of deionized water and 1 ml of ethanol. The obtained 913.32 mg (3 mmol) of sodium oleate was added to the reaction solution along with 1.057 ml (3 mmol) of oleic acid. The solution was heated to 393.15 K for 1 h and then heated with a heating rate of 2.5 K min^−1^ to reflux temperature (563.15 K), which was held for 1 h. The prepared nanoparticles were precipitated with ethanol three times and redispersed in hexane.

### 2.2. Transmission electron microscopy

Transmission electron microscopy (TEM) images were obtained by a ZEISS Leo 912 transmission electron microscope operated at an acceleration voltage of 120 kV. A diluted nanoparticle dispersion was casted onto a carbon-covered copper grid and the solvent was evaporated before measuring. Particle-size histograms were obtained by manual measurements of at least 300 nanoparticles and were refined according to a lognormal size distribution.

### 2.3. Energy-disperse X-ray

Energy-disperse X-ray (EDX) analysis was carried out on a Neon Zeiss 40 scanning electron microscope (SEM) operating at 5 kV acceleration voltage. The EDX spectra were measured at several varying sample positions and the resulting element composition was averaged from all measurements.

### 2.4. Powder X-ray diffraction

Powder X-ray diffraction (PXRD) was measured on a PANalytical X’Pert PRO diffractometer equipped with a secondary monochromator and a PIXcel detector using Cu *K*α radiation (λ = 1.54 Å). The samples were measured in the 2θ range of 5–80° with a step size of 0.026°. Rietveld refinement was carried out in the *FullProf* software (Rodríguez-Carvajal, 1993 ▸) using a pseudo-Voigt profile function. For averaged crystallite shapes, the spherical harmonics function describing the preferred orientation of crystallites was used (Bergmann *et al.*, 2001 ▸): 

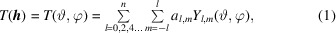

where ϑ and φ are polar and azimuthal angles describing the direction of the normal to the family of the lattice plane in a Cartesian coordinate system, *a* is the lattice parameter, and *Y* is the Lorentzian isotropic size broadening. The instrumental broadening was determined using an LaB_6_ reference (SR 660b, NIST).

### 2.5. Small-angle X-ray scattering

Small-angle X-ray scattering (SAXS) measurements were performed at the Gallium Anode Low-Angle X-ray Instrument (GALAXI) at JCNS, Forschungszentrum Jülich, Germany (Kentzinger *et al.*, 2016 ▸). Dilute nanoparticle dispersions in hexane were sealed in quartz capillaries and measured using a wavelength of λ = 1.3414 Å at two detector distances of 853 and 3548 mm, giving access to a *Q* range of 0.012 ≤ *Q* ≤ 0.3 Å^−1^. The data were recorded on a Pilatus 1M detector, radially averaged and normalized to absolute units using fluorinated ethylene propylene 1400 Å with a thickness of 0.35 mm as the reference material. Background scattering of the toluene solvent was subtracted.

### 2.6. Polarized small-angle neutron scattering

SANS was performed at the D22 instrument at Institut Laue–Langevin (ILL), Grenoble, France (Zákutná *et al.*, 2018 ▸). Dilute nanoparticle dispersions in *d*_8_-toluene were measured at ambient temperature and under a magnetic field of 1.4 T applied horizontally perpendicular to the neutron beam. Two instrument configurations were used with detector distances of 2 and 8 m, and collimations of 4 and 8 m, respectively, yielding a range in momentum transfer of 0.007 ≤ *Q* ≤ 0.2 Å^−1^. The incident neutron beam was polarized using a V-shaped supermirror polarizer. The efficiencies of the flipper and supermirror are 0.98 and 0.94, respectively, at a neutron wavelength of 7.21 Å. Data reduction was performed using the *Grasp* software (Dewhurst, 2003 ▸).

To model the core–shell superball morphology of a cuboidal nanoparticle with oleic acid ligand shell, we derived the scattering amplitude of the oriented core–shell superball according to

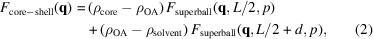

where the core–shell contrast is established by the scattering-length densities of nanoparticle core ρ_core_, shell ρ_OA_ (OA = oleic acid) and solvent ρ_solvent_. The scattering amplitude of the oriented superball *F*_superball_ with half the superball edge length *L*/2, the shell thickness *d* and the shape parameter *p* is given by Dresen *et al.* (2021 ▸). To obtain the orientationally averaged form factor, the oriented scattering amplitude was squared and subsequently integrated over all possible orientations and a lognormal size distribution, as detailed by Dresen *et al.* (2021 ▸).

### 2.7. Magnetization measurements

Magnetization measurements using vibrating sample magnetometry (VSM) were carried out on a Quantum Design PPMS Evercool II. Dispersions of the nanoparticles in toluene were sealed in glass ampoules. Isothermal magnetization was measured in a magnetic field range of up to ±9 T at 10 and 300 K. The magnetization *M*(*H*) (where *H* is magnetic field strength) at 298 K was evaluated according to the modified Langevin equation: 

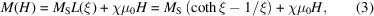

where *M*_S_ is the spontaneous magnetization and ξ = μμ_0_ *H*/*k*_B_ *T* is the Langevin parameter, with μ_0_ the permeability of free space, μ the integral particle moment, *k*_B_ the Boltzmann constant and *T* the temperature. The phenomenological susceptibility parameter χ accounts for the linear magnetization at high magnetic field, typically resulting from uncompensating diamagnetic contributions, *e.g.* from the sample holder or solvent.

### 2.8. Mössbauer spectroscopy

Mössbauer spectroscopy of ^57^Fe was carried out on a Wissel spectrometer in transmission geometry and using a proportional detector at ambient temperature without an external magnetic field. An α-Fe foil is used as standard, and spectrum fitting is carried out using the Wissel *NORMOS* routine (Brand *et al.*, 1983 ▸).

## 3. Results and discussion

### 3.1. Structure

Cobalt ferrite nanocubes were synthesized following a heating approach based on the decomposition of iron(III) acetylacetonate [Fe(acac)_3_] and cobalt(II) acetyltacetonate [Co(acac)_2_] precursors in benzyl ether (Wu *et al.*, 2014 ▸). To direct the particle growth towards nanocubes, equimolar amounts of oleic acid and sodium oleate were used as stabilizers. A surfactant-to-iron ratio of 3:0.8 was applied for all syntheses, aiming at a cubic morphology as reported for a ratio of at least 3:1 (Zeng *et al.*, 2004 ▸). For cobalt ferrite nanoparticles, a reduced amount of cobalt compared with the starting materials has been reported (Sathya *et al.*, 2016 ▸). A varying excess of cobalt with molar ratios of iron to cobalt of 2:1.15 (AH10), 2:1.3 (AH14) and 2:1.45 (AH15) was therefore applied for a systematic variation of the cobalt content.

**Figure 1:**
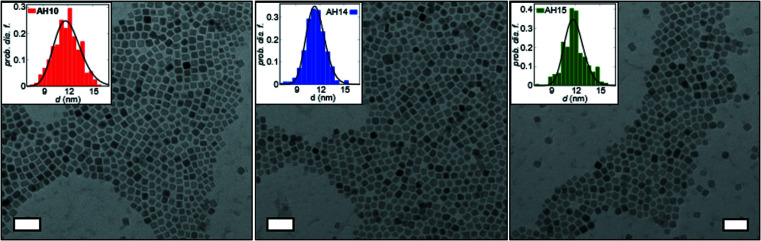
TEM bright-field micrographs of cobalt ferrite nanocubes. Inset: particle-size histograms with lognormal size distribution (lines). Scale bars: 50 nm.

TEM confirms a faceted morphology for all nanoparticle samples (Fig. 1 ▸). The mean particle edge lengths are very similar in the range of 11.6–12 nm, with size distributions of 13.6% (AH10) and 10% (AH14, AH15) as summarized in Table 1 ▸.

The superball form factor applied to SAXS data provides a means to quantify the cuboidal shape of nanoparticles in between that of a sphere and a perfect cube, where the shape parameter *p* = 1 corresponds to a sphere and *p* → ∞ for a perfect cube (Dresen *et al.*, 2021 ▸). Applied to the studied cobalt ferrite nanoparticles, a determined shape parameter of *p* = 1.7 indicates a clear cuboidal shape for all samples, with an even stronger cubicity for the AH10 sample (*p* = 2.4). The overall particle edge lengths and size distributions derived from SAXS (Fig. 4) are in general agreement with the TEM results.

The particle size and size distribution are hence comparable for these three samples of varying composition. SEM EDX analysis reveals global ratios of iron and cobalt of Fe:Co = 2:0.43 (AH10), 2:0.60 (AH14) and 2:0.57 (AH15).

The cobalt content in our samples is significantly lower than anticipated on the basis of the ratio of the starting materials, in agreement with earlier reports (Sathya *et al.*, 2016 ▸). However, the relative variation in cobalt content is still sufficient to study its influence on the intraparticle magnetization, in particular by direct comparison of AH10 and AH14.

**Figure 2:**
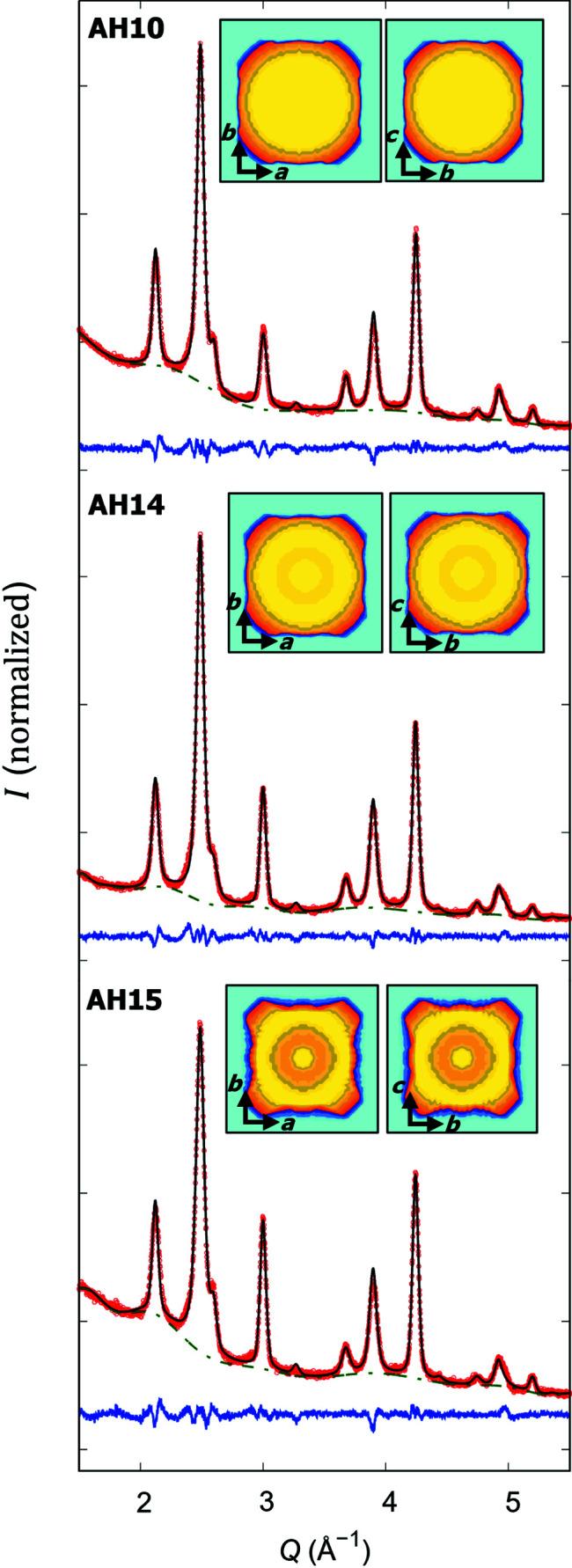
Rietveld refinements of PXRD patterns of AH10, AH14 and AH15 samples. The red dots, green dashed line, and black and blue lines correspond to the measured data, background, resulting fit and residuals, respectively. The small picture insets show the average density distribution in the different crystallographic directions.

The PXRD data (Fig. 2 ▸) are in line with a pure spinel crystal phase as expected for cobalt ferrite, without any evidence of impurities. All reflections are indexed according to the space group 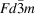 with lattice parameters ranging from 8.378 to 8.386 Å, *i.e.* in between the bulk lattice parameters of maghemite [*a* = 8.3500 Å; Boudeulle *et al.* (1983 ▸) or PDF2 No. 00-004-0755] and cobalt ferrite [*a* = 8.3919 Å; Natta & Passerini (1929 ▸) or PDF2 No. 00-022-1086], as indicated by the chemical composition.

The XRD data were refined by Rietveld analysis including the spherical harmonics function for an averaged crystalline shape and preferred orientation. As cobalt and iron are hard to distinguish using XRD, the Fe:Co ratio as determined using EDX analysis was distributed equally to the tetrahedral (A) and octahedral (B) sites of the spinel crystal structure. For a reasonable refinement, the AH14 and AH15 data sets required application of a strain model (Leineweber, 2011 ▸), yielding average maximum strain values of 27 (6) and 3.7 (9)%, respectively. These strain values, derived from the strain coefficients listed in Table 2 ▸, correspond to 1/4 of the apparent strain defined by Stokes & Wilson (1944 ▸) and represent the upper limit of the root mean square of variation in the lattice parameters across the sample (Robert & Novák, 2015 ▸). This indicates that the AH14 and AH15 samples have enhanced residual stress or non-uniform lattice distortion at the surface. This observation correlates with the lower degree of cubicity of these samples and might be attributed to enhanced strain in the rounded cubes corners.

The average coherent grain sizes determined by Rietveld refinements (Table 1 ▸) are in reasonable agreement with the particle sizes determined by TEM and SAXS.

Moreover, we observe a slightly larger isotropic displacement parameter for the B site compared with the A site. This may indicate a stronger degree of structural disorder on this site, in agreement with a stronger spin canting on the octahedral B site as observed for maghemite nanoparticles using nuclear-resonant scattering (Herlitschke *et al.*, 2016 ▸) and for cobalt ferrite nanoparticles using X-ray magnetic circular dichroism (Moya *et al.*, 2021 ▸).

### 3.2. Magnetism

**Figure 3:**
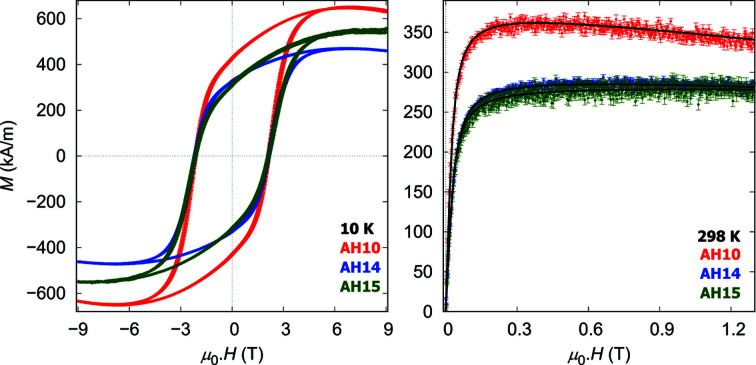
Magnetization curves of nanoparticle dispersions recorded at 10 and 298 K. Black lines represent Langevin fits.

Macroscopic magnetization measurements of dilute nanoparticle dispersions at ambient temperature (Fig. 3 ▸) reveal a pseudo-superparamagnetic behavior that allows one to determine the spontaneous magnetization and integral particle moment using Langevin analysis (Table 3 ▸). For all samples, the ratio of spontaneous magnetization and integral particle moment reveals a magnetic particle volume in agreement with the structural particle volume determined using TEM and SAXS analysis. We also observe a significantly increased spontaneous magnetization for the nanocubes with a lower Co^2+^ content, AH10, which approaches the bulk magnetization (466.4 kA m^−1^ for CoFe_2_O_4_, 473.8 kA m^−1^ for Fe_3_O_4_) (Schieber, 1967 ▸). With increasing molar amount of cobalt, a reduced spontaneous magnetization is observed in AH14 and AH15. This observation is in line with previous studies, which show a decrease of the spontaneous magnetization with increasing Co content (Sathya *et al.*, 2016 ▸; Salazar-Alvarez *et al.*, 2007 ▸; Torres *et al.*, 2015 ▸).

Magnetization measurements at low temperatures of 10 K reveal a strong coercivity for all samples that is not affected by the cobalt content (Fig. 3 ▸). The observed coercive field of 2.1 (1) T is larger than that reported for Co_*x*_Fe_3−*x*_O_4_ with 0.1 ≤ *x* ≤ 0.5 (Sathya *et al.*, 2016 ▸), as well as Co_0.6_Fe_2.4_O_4_ and Co_0.7_Fe_2.3_O_4_ (Wu *et al.*, 2014 ▸).

The nanoparticle size of all samples is below the critical size of cobalt ferrite for the single-domain state of 16 nm (Pal *et al.*, 2010 ▸). In this size range, surface effects typically become influential to the physical properties of nanoparticles and a reduced magnetization is often observed as a result of near-surface spin disorder. With its unique sensitivity to both structural and magnetic inhomogeneities on the nanoscale (Honecker *et al.*, 2022 ▸; Mühlbauer *et al.*, 2019 ▸), magnetic SANS is the technique of choice to unravel such surface-induced magnetization effects. Using polarized SANS techniques, a reduced magnetization near the particle surface is commonly found (Disch *et al.*, 2012 ▸; Krycka *et al.*, 2010 ▸) and its field dependence has recently been revealed (Zákutná *et al.*, 2020 ▸). At the same time, quantitative analysis can indicate a reduced magnetization throughout the nanoparticle interior (Krycka *et al.*, 2014 ▸; Disch *et al.*, 2012 ▸; Herlitschke *et al.*, 2016 ▸; Oberdick *et al.*, 2018 ▸), probably associated with structural disorder in the nanoscale crystals.

**Figure 4:**
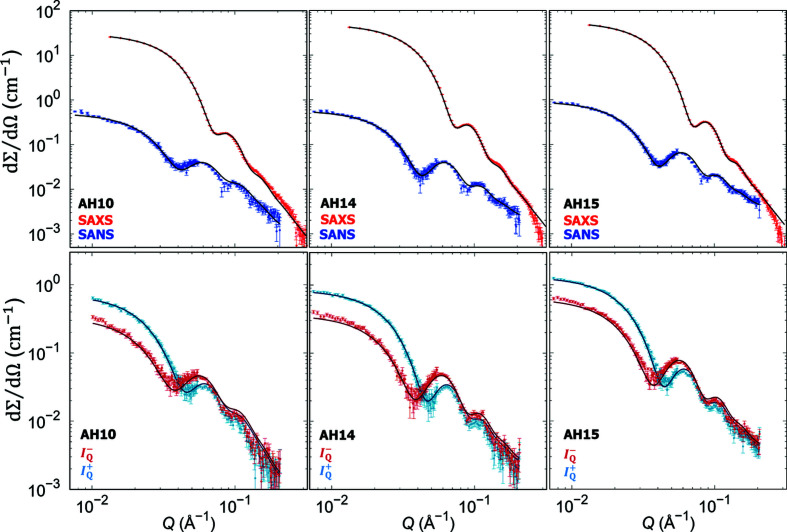
Small-angle scattering data for all samples with refinement of the superball chemical morphology (SAXS and nuclear SANS, top row) and magnetic contrast (polarized SANS, bottom row).

The AH14 and AH15 samples with higher amounts of cobalt also exhibit a non-negligible near-surface strain in our Rietveld analysis (Table 2 ▸). To rule out associated near-surface spin disorder as the origin of the reduced magnetization in these samples, magnetic SANS measurements were performed to elucidate the magnetization distribution within the nanoparticles with emphasis on a potential near-surface magnetization deviation. Nuclear and magnetic SANS results are presented in Fig. 4 ▸. Refinement of the chemical nanoparticle morphologies is based on the purely nuclear SANS scattering cross sections, determined from sector cuts in the direction parallel to a saturating magnetic field (1.4 T). The nuclear core–shell superball form-factor model was constrained to the edge length, size distribution and shape parameter obtained using SAXS. The only structural parameter based on SANS, the thickness of the oleic acid ligand shell is in the range of *d* = 1.3–1.5 nm, in excellent agreement with previous refinements of oleic acid capped ferrite nanoparticles (Disch *et al.*, 2012 ▸; Herlitschke *et al.*, 2016 ▸; Zákutná *et al.*, 2020 ▸). Scattering-length densities of the cobalt ferrite were calculated from the chemical composition and the mass density derived from the lattice parameters (PXRD results, Table 2 ▸) and were kept constant during refinement (ρ_AH10_ = 6.450 × 10^−6^ Å^−2^, ρ_AH14_ = 6.363 × 10^−6^ Å^−2^, ρ_AH15_ = 6.474 × 10^−6^ Å^−2^). The values of scattering-length densities of the solvent (ρ_*d*-toluene_ = 5.66 × 10^−6^ Å^−2^) and oleic acid surfactant (ρ_OA_ = 0.078 × 10^−6^ Å^−2^) were also kept constant. Additionally, a contribution of excess oleic acid micelles with a radius of *r*_OA_ = 2.1 nm was considered. The refined parameters of the superball form factor are summarized in Table 4 ▸.

With the chemical nanoparticle morphology fixed, the magnetization distribution was refined using the difference between the neutron-spin-dependent scattering cross sections *I* ^+^ and *I* ^−^ extracted perpendicular to the applied magnetic field, which scales with the nuclear and magnetic form-factor amplitudes. The particle-size distribution and the shape parameter were constrained to be equal for both magnetic and nuclear form factors, leaving only the magnetic superball edge length and the magnetic scattering cross section as parameters for the polarized SANS fit.

The magnetic SANS data for all samples are best described with the magnetic superball edge length equal to the nuclear edge length, indicating the absence of enhanced disorder from canted or randomly oriented (disordered) spins at the nanoparticle surface, which would resemble a magnetic core–shell structure. This observation supports the high crystallinity of prepared nanoparticles as observed from PXRD, where the coherent domain size is close to the whole particle size, and suggests that the observed strain coefficients do not affect the near-surface magnetization.

The derived magnetic scattering-length densities relate to the intraparticle magnetization according to

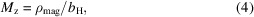

where *b*_H_ = 2.91 × 10^8^ A^−1^m^−1^ is the magnetic scattering length. The magnetization obtained using magnetic SANS, and the integral particle moment related to the nanoparticle volume by μ = *M*_S_ *V*, are in excellent agreement with the macroscopic magnetization (Table 3 ▸), confirming consistency of our results.

The variation in magnetization observed both macroscopically and by magnetic SANS analysis is attributed to the presence of Co^2+^ ions in both octahedral and tetrahedral sites, where this mixed occupancy can destabilize the ferrimagnetic order of the Fe^3+^ ions of the cobalt-free magnetite/maghemite (Moya *et al.*, 2021 ▸). This means that the magnetic moment per unit cell reduces with increasing Co^2+^ distribution inside the spinel structure. This is supported by our polarized SANS and VSM results, which consistently reveal decreasing spontaneous magnetization with increasing cobalt content.

All presented samples exhibit a high crystallinity and homogeneous Fe/Co distribution, which is supported by our SANS results that give no indication of a magnetic core–shell structure or enhanced near-surface spin disorder within the accuracy of the analysis. The main prospect for CoFe_2_O_4_ is that the enhanced orbital moment of Co^2+^ in the spinel lattice improves the magnetocrystalline anisotropy compared with the maghemite/magnetite structure (Wolf, 1957 ▸; Slonczewski, 1958 ▸). In combination with the single-domain particle size, good crystallinity and shape anisotropy arising from the cuboidal nanoparticle morphology, the strong uniaxial anisotropy accounts for a high magnetic hardness of 2.1 T, as observed in our samples.

**Figure 5:**
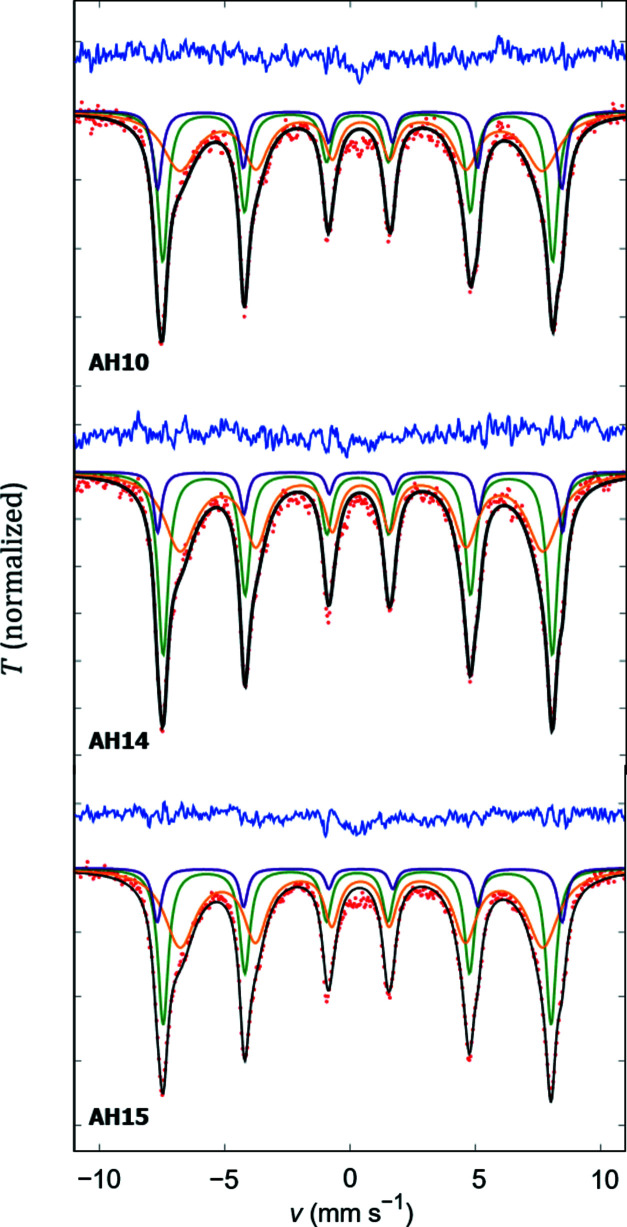
Mössbauer spectroscopy at room temperature. Red points and black and blue lines represent the measured data, fit and residuals, respectively. Individual sextet subspectra of (Fe^3+^)_A_, [Fe^3+^]_B_ and [Fe^2+^]_B_ are shown as green, orange and violet lines, respectively.

To fully unravel the iron occupancy and correlate it with the magnetic properties of our cuboidal nanoparticles, Mössbauer spectroscopy measurements were performed (Fig. 5 ▸). The Mössbauer spectra are consistently described by a superposition of three sextet subspectra, which correspond to the magnetically ordered states of iron atoms. The Mössbauer spectroscopy results are shown in Table 5 ▸. The sextet with the smallest isomer shift corresponds to the Fe^3+^ in the tetrahedral sites (Fe^3+^)_A_. The second sextet with larger isomer shift is best described using a hyperfine field distribution due to the non-equivalent octahedral sites corresponding to Fe^3+^ in octahedral sites (Roca *et al.*, 2007 ▸). The variation in octahedral sites may be related to enhanced structural disorder on the octahedral site, in agreement with earlier results on maghemite nanoparticles (Herlitschke *et al.*, 2016 ▸). The third sextet corresponds to a small amount of Fe^2+^ in the octahedral sites, which has the largest isomer shift due to the stronger shielding effect from the *s* electron. The fit results of the three subspectra indicate that the non-exchanging iron, (Fe^3+^)_A_, exhibits a constant isomer shift for all samples, whereas for the double-exchanging iron, [Fe^3+^]_B_, a clear variation of the isomer shift is visible, correlated with an increasing amount of Fe^3+^ in this site.

In the magnetite structure, the Fe^2+^ sub-spectrum is typically not detected by Mössbauer spectroscopy due to the fast electron-hopping process between octahedral sites of Fe^2+^ and Fe^3+^, leading to a mixed subspectrum with an average oxidation state of Fe^2.5+^ and a typical isomer shift of 0.66 mm s^−1^ (Daniels & Rosencwaig, 1969 ▸; Vandenberghe *et al.*, 2000 ▸). However, as the presence of cobalt on the octahedral site eliminates part of the interactions between Fe^2+^ and Fe^3+^, the extra sextet from Fe^2+^ is visible here, along with a reduced isomer shift of the [Fe^3+^]_B_ site. The area under the subspectra is directly proportional to the number of Fe atoms in A and B sites. The ratio between B and A site occupancy of Fe^3+^ is 1.16, 1.23 and 1.45 for AH10, AH14 and AH15, respectively, in between those expected for a maghemite structure (B/A = 1.667) and an ideal magnetite structure (B/A = 1) (Vandenberghe *et al.*, 2000 ▸; Roca *et al.*, 2007 ▸).

From the ^57^Fe cation distribution in the spinel structure and the elemental composition determined from SEM EDX, the inversion parameter can be extracted. The distribution of the Co^2+^ cations is estimated from the charge balance, with the atomic fraction obtained from EDX analysis. The obtained distributions of the cations from Mössbauer refinements are ( 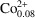 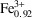 )_A_[ 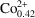 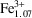 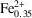 ]_B_O_4_, ( 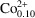 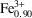 )_A_[ 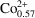 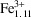 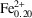 ]_B_O_4_ and ( 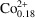 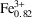 )_A_[ 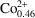 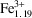 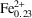 ]_B_O_4_ for AH10, AH14 and AH15 samples, respectively. The inversion parameter is defined as the number of Fe^3+^ cations in the tetrahedral sites leading to the values of 0.92, 0.90 and 0.82 for AH10, AH14 and AH15 samples, respectively.

We therefore conclude that with increasing amount of cobalt in the material, a larger B-site occupancy is observed for Fe^3+^, corresponding to a preferential occupancy of the A site by cobalt and, in consequence, a reduction of the degree of spinel inversion. At the same time, the Mössbauer interpretation is biased towards a maghemite-like structure with increasing amount of cobalt.

## 4. Conclusions

Monodisperse cuboidal nanoparticles of cobalt ferrite with very similar particle size, but distinct sub-stoichiometric Co concentration, were synthesized and structurally and magnetically characterized using a combination of atomistic and nanoscale-sensitive techniques.

We observed a slight variation in cubicity, quantified by the superball shape parameter, that correlates with a larger surface strain for the less cubic samples, AH14 and AH15. Apart from this, all samples exhibit a high crystallinity, with a crystalline coherent particle size in good agreement with the overall particle size. Magnetic SANS indicates a very homogeneous magnetization distribution throughout the particles, without any indication of enhanced spin disorder near the particle surface. As a result, we observed a high coercivity of 2.1 T at 10 K.

The Co content further correlates with a significant reduction in magnetization, observed simultaneously by both magnetic SANS and macroscopic VSM measurements. On the atomistic scale, the combination of Mössbauer spectroscopy and EDX enables analysis of the detailed site occupancies, revealing a preferential occupancy of cobalt in the tetrahedral site and a corresponding reduction of the degree of spinel inversion with increasing cobalt content.

These results demonstrate a plateau of high magnetocrystalline anisotropy and resulting strong coercivity unaffected by Co concentration, with the potential to adjust the spontaneous magnetization against magnetocrystalline anisotropy and coercivity scaling with Co concentration. 

## Figures and Tables

**Table 1:** Nanoparticle morphology as determined from TEM, SAXS and XRD, including the mean particle edge lengths *L* with the lognormal size distribution σ and the superball shape parameter *p*

Sample	AH10	AH14	AH15
*L*_TEM_ (nm)	12.0 (1)	11.6 (1)	11.9 (1)
σ_TEM_ (%)	13.6 (9)	10.0 (2)	9.7 (1)
			
*L*_SAXS_ (nm)	10.08 (4)	10.46 (2)	10.88 (2)
σ_SAXS_ (%)	13.0 (1)	12.2 (1)	11.5 (7)
*p*	2.39 (8)	1.71 (3)	1.67 (2)
			
*L*_XRD_ (nm)	11.6 (1.4)	11.2 (1.2)	11.4 (2.0)

**Table 2:** Rietveld refinement results including lattice parameter *a*, fractional position of the oxygen site *u*, isotropic displacement parameters *B*_iso_, Lorentzian isotropic size broadening *Y*, Gaussian isotropic size broadening GausSiz, coefficients of spherical harmonics *K*_*lm*_ and coefficients of the strain model *S*_*hkl*_

	AH10	AH14	AH15
Space group	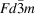		
*a* (Å)	8.3777 (3)	8.3837 (4)	8.3860 (4)
*u* (*x*, *y*, *z*)	0.2494 (2)	0.2599 (1)	0.2610 (14)
			
Profile function	Thompson–Cox–Hastings pseudo-Voigt		
*B*_iso_ A site (Å^−2^)	2.29 (5)	0.057 (1)	0.78 (5)
*B*_iso_ B site (Å^−2^)	2.72 (3)	7.42 (5)	6.57 (5)
*Y* (0.01°)	0.429 (6)	0.415 (7)	0.378 (7)
GausSiz (0.01°)	0.346 (3)	0.284 (4)	0.244 (4)
Zero shift (0.01°)	0.003 (2)	0.012 (2)	0.013 (3)
*R*_f_ (%)	2.62	2.62	2.73
*R*_B_ (%)	3.07	2.15	2.36
*R*_wp_ (%)	2.84	4.24	3.58
*R*_exp_ (%)	1.93	2.17	2.04
χ^2^	2.16	3.81	3.08
			
Spherical harmonics	Laue class *m*3*m*		
*K*_00_	0.0	0.0	0.0
*K*_41_	4.8 (2)	6.0 (1)	12.1 (2)
*K*_61_	−0.2 (1)	−1.2 (1)	−4.4 (1)
*K*_62_	0.0	0.0	0.0
*K*_81_	−1.8 (1)	−2.6 (1)	−6.6 (1)
			
Strain model	Laue class *m*3*m*		
*S*_400_	–	–	–
*S*_220_	–	−1.7 (1)	3.7 (1)
			
Background function	Chebyshev polynomial function		
Number of coefficients	22	21	19
			
Total fit parameters	32	33	31

**Table 3:** Nanoparticle magnetization determined from macroscopic magnetization and magnetic SANS data Spontaneous magnetization *M*_S_, integral particle moment μ and coercive field at low temperature μ_0_ *H*_C_ are listed.

	AH10	AH14	AH15
Macroscopic magnetization			
*M*_S_ (kA m^−1^)	387.0 (2)	299.0 (3)	286.1 (6)
μ (10^4^μ_B_)	3.52 (4)	2.82 (2)	3.07 (3)
μ_0_ *H*_C_ (T) at 10 K	2.1 (1)	2.1 (1)	2.1 (1)
			
Magnetic SANS			
*M*_S_ (kA m^−1^)	347 (7)	289 (3)	296 (3)
μ (10^4^μ_B_)	3.28 (6)	2.72 (3)	3.09 (4)

**Table 4:** Refinement results of the nanoparticle morphology from SAXS, nuclear SANS and magnetic SANS, including the mean particle edge lengths *L* with the lognormal size distribution σ, the superball shape parameter *p*, the scale factors of the nanoparticle *I*_0_ and excess oleic acid *I*_0,OA_ contributions, and a background parameter bgr Parameters not mentioned in nuclear and magnetic SANS were kept fixed as determined using SAXS and nuclear SANS, respectively.

	AH10	AH14	AH15
SAXS			
*L* (nm)	10.08 (4)	10.46 (2)	10.88 (2)
σ (%)	13.0 (1)	12.2 (1)	11.5 (7)
*p*	2.39 (8)	1.71 (3)	1.67 (2)
*I*_0_ (Å^−2^cm^−1^)	0.0256 (1)	0.04214 (6)	0.04005 (8)
*V*_SAXS_ (10^−25^ m^−3^)	8.77	8.75	9.68
χ^2^	13.8	41	8
			
Nuclear SANS			
*d* (nm)	1.47 (5)	1.26 (4)	1.45 (4)
*I*_0_ (Å^−2^ cm^−1^)	0.0181 (2)	0.03752 (3)	0.0370 (3)
*I*_0, OA_ (Å^−2^ cm^−1^)	0.43 (1)	0.21 (1)	0.35 (2)
bgr (cm^−1^)	0.0011 (2)	0.0017 (2)	0.0034 (2)
χ^2^	4.0	3.2	4.5
			
Magnetic SANS			
ρ_mag_ (10^−6^ Å^−2^)	1.01 (2)	0.84 (1)	0.86 (1)
χ^2^	4.0	2.4	6.8

**Table 5:** Mössbauer spectroscopy results including isomer shift δ, hyperfine field *B*_hf_, mass fraction *w* and line width Γ for three subspectra of each sample

Sample	Site	δ (mm s^−1^)	*B*_hf_ (T)	*w* (%)	Γ (mm s^−1^)
AH10	[Fe^2+^]_B_	0.387 (9)	50.05 (9)	14.98	0.52 (3)
	[Fe^3+^]_B_	0.425 (14)	44.78 (35)†, 51.8‡	45.67	0.54 (5)
	(Fe^3+^)_A_	0.292 (5)	48.28 (6)	39.35	0.33 (4)
					
AH14	[Fe^2+^]_B_	0.406 (9)	50.16 (5)	9.15	0.51 (2)
	[Fe^3+^]_B_	0.435 (9)	44.89 (20)†, 50.9‡	50.20	0.57 (4)
	(Fe^3+^)_A_	0.296 (4)	48.16 (4)	40.65	0.30 (5)
					
AH15	[Fe^2+^]_B_	0.415 (6)	50.24 (5)	10.13	0.50 (1)
	[Fe^3+^]_B_	0.443 (6)	44.97 (13)†, 50.7‡	53.17	0.58 (2)
	(Fe^3+^)_A_	0.295 (2)	48.11 (2)	36.70	0.33 (3)

† Average *B*_hf_ values given for [Fe^3+^]_B_.

‡ Maximum *B*_hf_ values given for [Fe^3+^]_B_.
